# *In Vivo* Molecular MRI Imaging of Prostate Cancer by Targeting PSMA with Polypeptide-Labeled Superparamagnetic Iron Oxide Nanoparticles

**DOI:** 10.3390/ijms16059573

**Published:** 2015-04-28

**Authors:** Yunkai Zhu, Ying Sun, Yaqing Chen, Weiyong Liu, Jun Jiang, Wenbin Guan, Zhongyang Zhang, Yourong Duan

**Affiliations:** 1 Department of Ultrasound in Medicine, Xinhua Hospital Affiliated to Shanghai Jiaotong University School of Medicine, Shanghai 200092, China; E-Mails: tttmax2004@126.com (Y.Z.); lwy_ultras@126.com (W.L.); tenine@163.com (J.J.); 2 State Key Laboratory of Oncogenes and Related Genes, Shanghai Cancer Institute, Renji Hospital Affiliated to Shanghai Jiaotong University School of Medicine, Shanghai 200032, China; E-Mail: ysun@shsci.org; 3 Department of Pathology, Xinhua Hospital Affiliated to Shanghai Jiaotong University School of Medicine, Shanghai 200092, China; E-Mail: celiceli02@126.com; 4 Department of Radiology, Xinhua Hospital Affiliated to Shanghai Jiaotong University School of Medicine, Shanghai 200092, China; E-Mail: tttmax2004@hotmail.com

**Keywords:** molecular imaging, MRI, SPIONs, polypeptide, prostate neoplasms, PSMA

## Abstract

The prostate specific membrane antigen (PSMA) is broadly overexpressed on prostate cancer (PCa) cell surfaces. In this study, we report the synthesis, characterization, *in vitro* binding assay, and* in vivo* magnetic resonance imaging (MRI) evaluation of PSMA targeting superparamagnetic iron oxide nanoparticles (SPIONs). PSMA-targeting polypeptide CQKHHNYLC was conjugated to SPIONs to form PSMA-targeting molecular MRI contrast agents. *In vitro* studies demonstrated specific uptake of polypeptide-SPIONs by PSMA expressing cells. *In vivo* MRI studies found that MRI signals in PSMA-expressing tumors could be specifically enhanced with polypeptide-SPION, and further Prussian blue staining showed heterogeneous deposition of SPIONs in the tumor tissues. Taken altogether, we have developed PSMA-targeting polypeptide-SPIONs that could specifically enhance MRI signal in tumor-bearing mice, which might provide a new strategy for the molecular imaging of PCa.

## 1. Introduction

Prostate cancer (PCa) is the most common malignancy affecting men in the Western world [1]. Transrectal ultrasound, magnetic resonance imaging (MRI), and nuclear medicine is currently used in PCa imaging [2]. However, as current imaging modalities provide limited functional information of PCa, the sensitivity of these imaging modalities remains relatively poor [3], leading to under-treatment of the aggressive disease. On the other hand, the presence of chronic prostatitis is often indistinguishable from PCa lesions with current imaging modalities [4], subsequently resulting in unnecessary biopsies [5]. Therefore, improvements in current imaging modalities are urgently needed.

Molecular imaging enables the visualization of tumor-specific biomarkers involved in the development and progression of tumors [6]. By designing imaging probes offering specific bindings with tumor biomarkers, tumor lesions could be detected in their early stages. More importantly, benign tissues are not enhanced due to their negative expression of tumor biomarkers. Therefore, the accuracy of PCa imaging might be significantly improved with molecular imaging.

Prostate specific membrane antigen (PSMA), a transmembrane 750-amino-acid type II glycoprotein, is a promising biomarker of PCa. PSMA is markedly overexpressed on PCa cell membranes, and its expression has also been found to increase with tumor grade and stage [7,8]. In addition, the cytoplasmic domain of PSMA mediates internalization and endosomal recycling, which leads to high intracellular concentration of PSMA-targeting imaging probes [9]. All these properties have caused PSMA-targeting probes to be the research focus in PCa imaging.

MRI provides excellent soft tissue contrast and multidimensional information, which is important in PCa localization and prostate biopsy guidance. In recent years, multiple groups have been actively pursuing the development of novel molecular contrast agents for MRI. Among these contrast agents, superparamagnetic iron oxide nanoparticles (SPIONs) have been a major area of research due to their excellent negative contrast of PCa lesion under the high signal background of adjacent benign tissues [10,11,12]. Moreover, SPIONs can be readily bound to various molecular markers, including ligands, antibodies, polypeptides, and aptamers [13]. In most previous studies, antibodies were employed for the fabrication of PSMA-targeting SPIONs [14,15]. In addition to intact antibodies, small molecules can also be conjugated to SPIONs surfaces without compromising the affinity [16]. In this work, PSMA-targeting SPIONs are synthesized with PSMA-targeting polypeptide (CQKHHNYLC, C_1_–C_9_ disulfide) for further* in vivo* MRI imaging of PCa xenograft tumor.

## 2. Results

### 2.1. Characterization of Polypeptide-SPIONs

**Figure 1:**
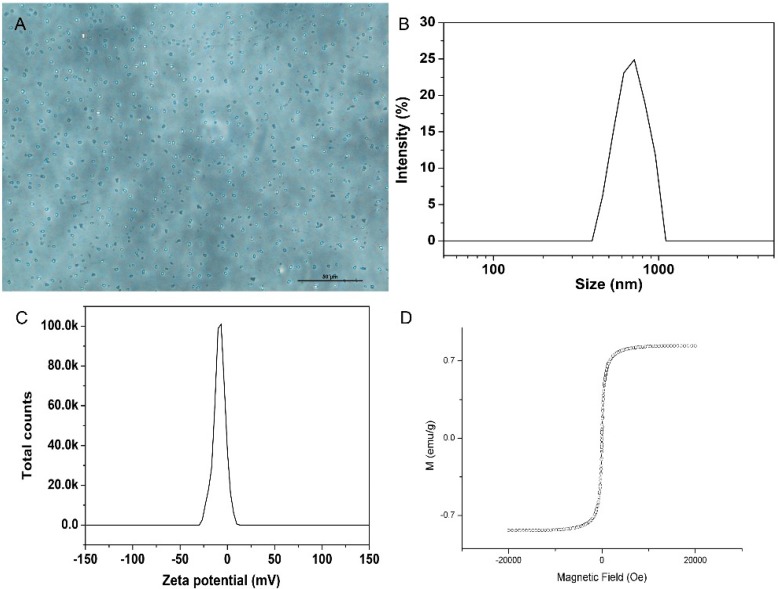
Characterization of polypeptide-SPIONs. (**A**) Light microscope of polypeptide-SPIONs (×400); (**B**) Size distribution of polypeptide-SPIONs; (**C**) Zeta potential of polypeptide-SPIONs; (**D**) VSM curve of polypeptide-SPIONs.

Inverted microscope demonstrated that the polypeptide-SPIONs exhibited a smooth and uniform spherical morphology (Figure 1A). The size distribution was in the range of 600–800 nm with a zeta potential of −20.5 mV (Figure 1B,C). The VSM results confirmed the superparamagnetic characteristic of polypeptide-SPIONs (Figure 1D).

### 2.2. In Vitro MRI of Polypeptide-SPIONs

**Figure 2:**
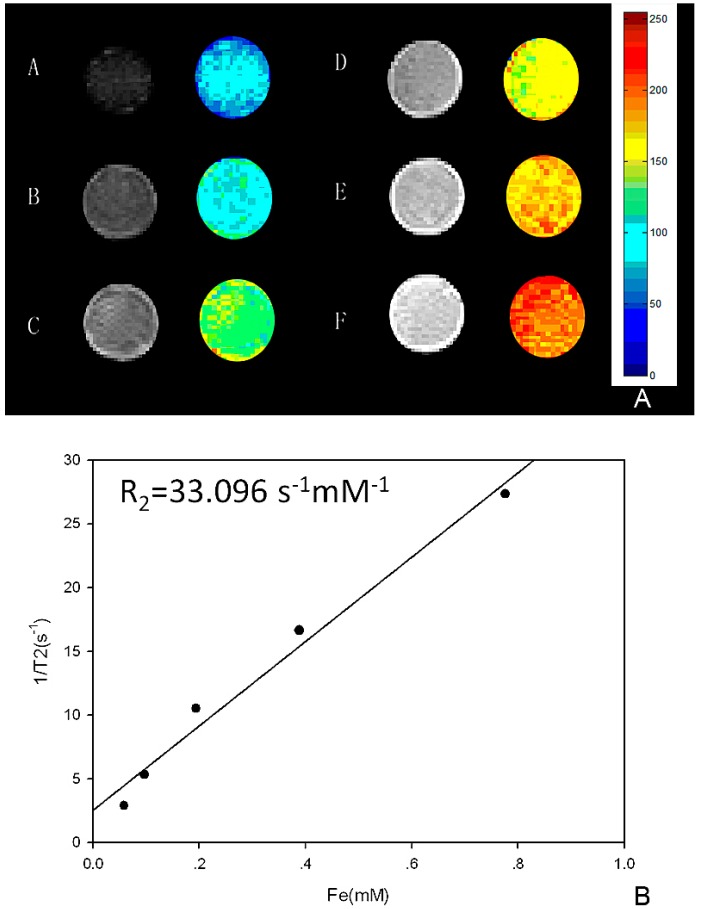
*In vitro* MRI of polypeptide-SPIONs. (**A**)* In vitro* MRI images of polypeptide-SPIONs: (**A**–**E**) polypeptide-SPIONs with Fe_3_O_4_ concentration 0.060, 0.030, 0.015, 0.007, and 0.0045 mg/mL, respectively; **F**: de-ionized water; (**B**) The correlation between the Fe_3_O_4_ concentration and 1/T2.

T2 weighted images of polypeptide-SPIONs are shown in Figure 2. The darkening effect of polypeptide-SPIONs increased with Fe_3_O_4_ concentration (Figure 2A). A significant linear fit was obtained between the Fe_3_O_4_ concentration and the 1/T2 with *R*_2_ = 33.096 s^−1^·mM^−1^ (Figure 2B).

### 2.3. In Vitro Binding Assay

**Figure 3:**
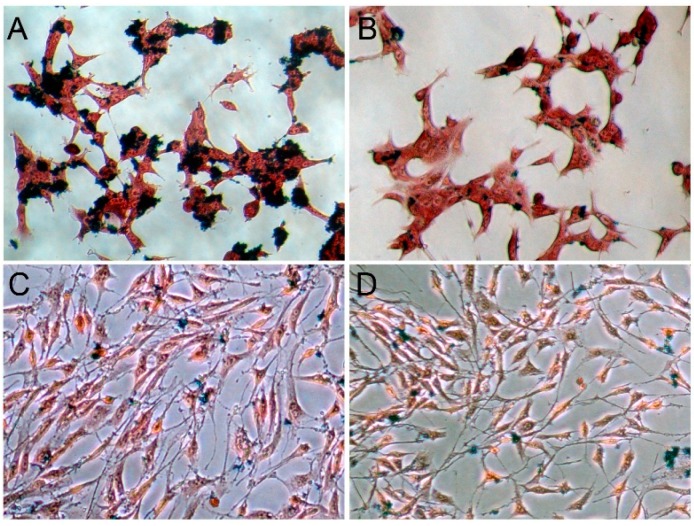
*In vitro* Prussian blue staining results (×200). (**A**,**B**) Prussian blue staining of LNCaP cells incubated with polypeptide-SPIONs and non-targeted SPIONs, respectively; (**C**,**D**) Prussian blue staining of PC3 cells incubated with polypeptide-SPIONs and non-targeted SPIONs, respectively.

Prussian blue staining showed greater iron uptake by LNCaP cells (PSMA positive) when incubated with polypeptide-SPIONs (Figure 3A) than with non-targeted SPIONs alone (Figure 3B), confirming that conjugation of SPIONs to polypeptide facilitated their uptake by the LNCaP cells. No significant iron uptake was observed for PC3 cells (PSMA negative) when incubated with polypeptide-SPIONs or non-targeted SPIONs (Figure 3C,D).

### 2.4. In Vivo MRI

**Figure 4:**
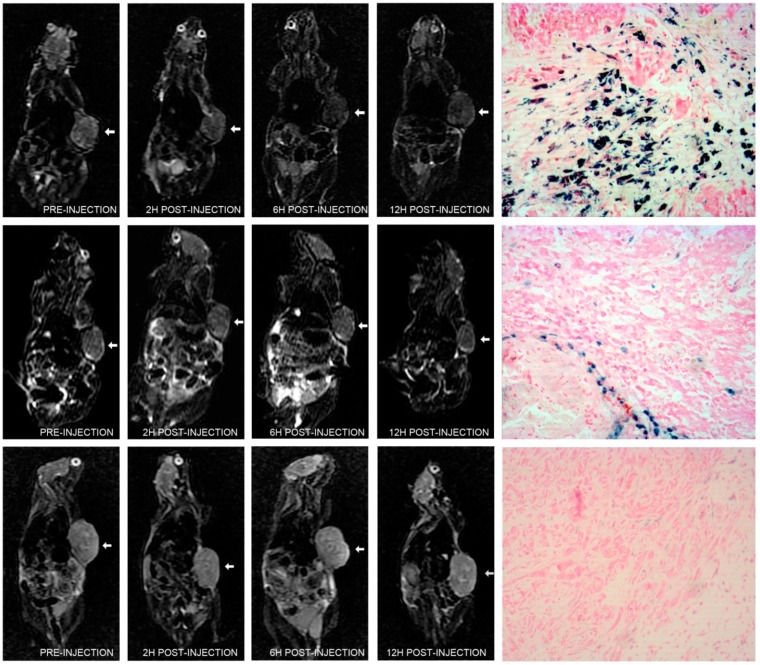
*In vivo* MRI images and histologic results. (**Top panel**) T2 weighted images of LNCaP tumor (arrow) pre-injection, different time point post-injection of polypeptide-SPIONs (Fe_3_O_4_, 0.240 mg/mL) and corresponding Prussian blue staining results (×100); (**Middle panel**) T2 weighted images of LNCaP tumor (arrow) pre-injection, different time point post-injection of non-targeted SPIONs (Fe_3_O_4_, 0.240 mg/mL) and corresponding Prussian blue staining results (×100); (**Bottom panel**) T2 weighted images of PC3 tumor (arrow) pre-injection, different time point post-injection of polypeptide-SPIONs (Fe_3_O_4_, 0.240 mg/mL) and corresponding Prussian blue staining results (×100).

**Figure 5:**
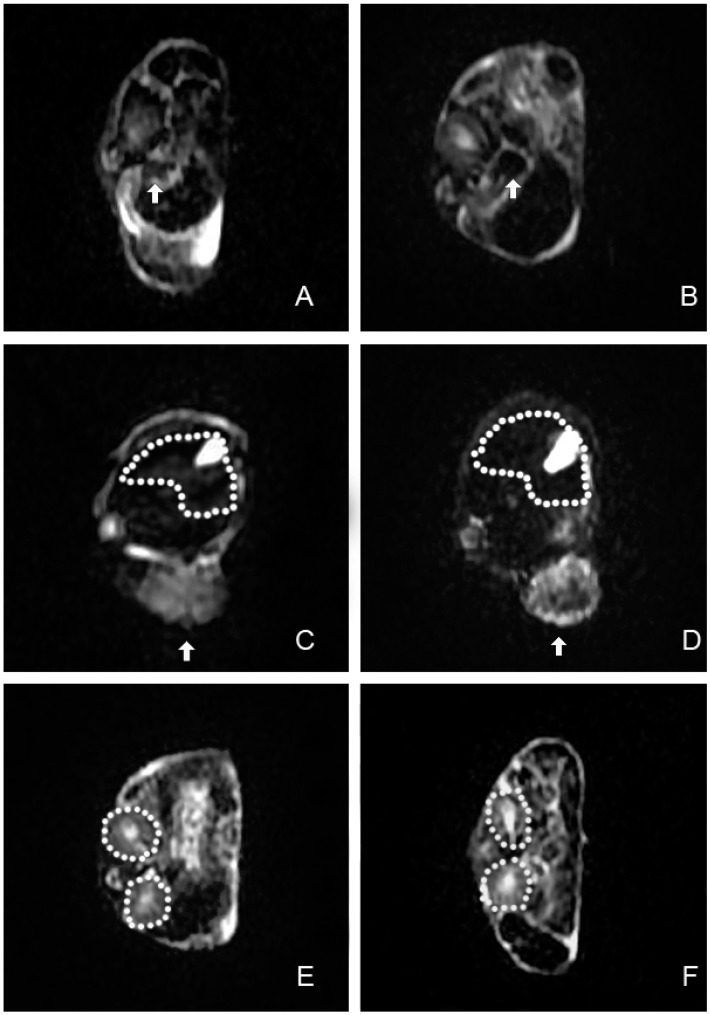
*In vivo* MRI images of the spleen, liver, and kidney. (**A**,**B**) T2 weighted images of the spleen (arrows) before SPIONs injection and 6 h post-injection; (**C**,**D**) T2 weighted images of the liver (dash line) before SPIONs injection and 6 h post-injection. Arrows indicated LNCaP xenograft tumor; (**E**,**F**) T2 weighted images of the kidney (dash line) before SPIONs injectionand 6 h post-injection.

The well-being (reflex, alertness, breathing, feces, and urine) of all mice, post-injection, was not changed. In LNCaP tumor-bearing mice injected with polypeptide-SPIONs, T2 signal reduction within tumors was observed 6–12 h post-injection in all concentration subgroups, along with 2 h post-injection of polypeptide-SPIONs with Fe_3_O_4_ concentration of 0.240 mg/mL (Figure 4). No clearly appreciable tumor signal changes were observed in the control groups at any of the three post-contrast time points (Figure 4). Following the injection of SPIONs, obvious negative contrast was observed in the spleen. Only slightly negative contrast was seen in the liver (Figure 5). In contrast, no signal reduction was found in the kidney after the injection of SPIONs.

The quantitative analyses of* in vivo* MRI images of all subgroups were concluded in Table 1 and Figure S1 as RSE data. LNCaP tumor-bearing mice injected with polypeptide-SPIONs demonstrated marked increase of RSE 6–12 h post-injection compared with 2 h post-injection (*p* < 0.05). The highest RSE was observed 6 h post-injection. Twelve hours post-injection, the RSE slightly decreased, but no differences were found compared with the RSE of 6 h (*p* > 0.05). The results also revealed that there was a trend toward increasing RSE with increasing polypeptide-SPIONs concentration. The highest RSE was observed 6 h post-injection with Fe_3_O_4_ concentration of 0.240 mg/mL. Compared with LNCaP tumor-bearing mice injected with polypeptide-SPIONs, the control group had a lower RSE. The highest RSE was 12.0 ± 2.0 and 11.1 ± 3.1 respectively for LNCaP tumor-bearing mice injected with non-targeted SPIONs and PC3 tumor-bearing mice injected with polypeptide-SPIONs. Moreover, no significant difference was found between the RSE of different post-contrast time points in the control group.

**Table 1:** RSE data in LNCaP tumor bearing mice injected with polypeptide-SPIONs, non-targeted SPIONs, and PC3 tumor bearing mice injected with polypeptide-SPIONs.

Post Injection Time	LNCaP + Polypeptide-SPIONs (Fe_3_O_4_ 0.240 mg/mL)	LNCaP + Polypeptide-SPIONs (Fe_3_O_4_ 0.120 mg/mL)	LNCaP + Polypeptide-SPIONs (Fe_3_O_4_ 0.060 mg/mL)	LNCaP + Polypeptide-SPIONs (Fe_3_O_4_ 0.030 mg/mL)	LNCaP + Non-Targeted SPIONs (Fe_3_O_4_ 0.240 mg/mL)	PC3 + Polypeptide-SPIONs (Fe_3_O_4_ 0.240 mg/mL)	*p*-Value
2 h	19.9 ± 3.4 *	15.7 ± 2.7 *	11.8 ± 1.6	11.3 ± 1.4	9.5 ± 2.0	8.7 ± 2.2	0.000
6 h	34.3 ± 3.6 *	31.1 ± 2.7 *	23.5 ± 1.4 *	19.6 ± 0.9 *	11.9 ± 3.9	11.1 ± 3.1	0.000
12 h	30.9 ± 1.4 *	30.0 ± 1.4 *	22.8 ± 1.8 *	18.5 ± 1.5 *	12.0 ± 2.0	10.4 ± 2.6	0.000
*p*-Value	0.000	0.000	0.000	0.000	0.304	0.179	

* Compared with control groups, *p* < 0.05. LNCaP, human prostate adenocarcinoma cells derived from the lymph node metastasis; SPION, superparamagnetic iron oxide nanoparticles; PC3, human prostate adenocarcinoma cells derived from the bone metastasis.

### 2.5. Histologic Analysis

The presence of PCa was histologically confirmed in all excised tumors. None of the tumors in this study were hemorrhagic. Immunohistochemistry demonstrated elevated PSMA expression within LNCaP tumors and negative PSMA expression within PC3 tumors. In LNCaP tumor-bearing mice injected with polypeptide-SPIONs, Prussian blue staining showed heterogeneous deposition of SPIONs in the tumor tissues (Figure 4). Further quantification analyses of Prussian blue staining were concluded in Table 2. We found a correlation between RSE and Prussian blue labeling density (*r* = 0.755, *p* = 0.000, Figure S2). In contrast, only a small amount of SPION uptake was observed in the control group (Figure 4). The Prussian blue labeling density was 3.2 ± 2.2 and 1.4 ± 0.6, respectively, for LNCaP tumor-bearing mice injected with non-targeted SPIONs and PC3 tumor-bearing mice injected with polypeptide-SPIONs, which was significantly lower than LNCaP tumor-bearing mice injected with polypeptide-SPIONs (*p* < 0.05).

**Table 2:** The Prussian blue labeling density in LNCaP tumor bearing mice injected with polypeptide-SPIONs.

Histologic Results	Fe_3_O_4_ 0.240 mg/mL	Fe_3_O_4_ 0.120 mg/mL	Fe_3_O_4_ 0.060 mg/mL	Fe_3_O_4_ 0.030 mg/mL	*p*-Value
Prussian blue labeling density	11.2 ± 2.1	9.7 ± 1.4	8.0 ± 1.3	6.9 ± 1.0	0.002

**Figure 6:**
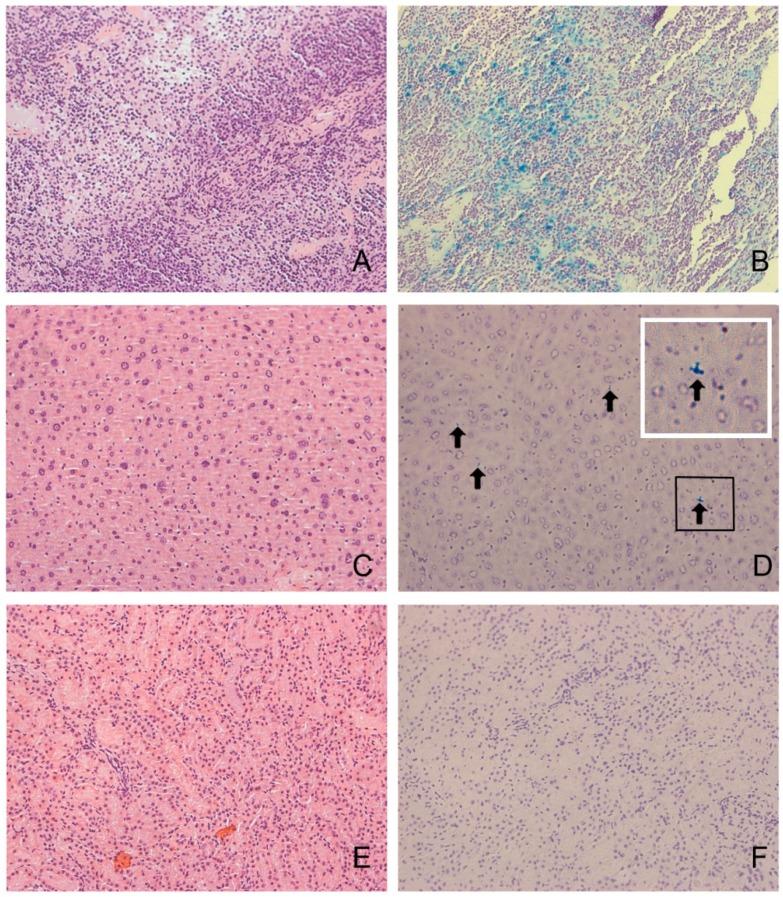
Histologic results of the spleen, liver, and kidney (×200). (**A**,**B**) HE staining and Prussian blue staining of the spleen. High levels of iron uptake were observed in the spleens; (**C**,**D**) HE staining and Prussian blue staining of the liver. A low level of iron uptake (arrows) was seen in the liver. White line box is the enlarged picture of black line box; (**E**,**F**) HE staining and Prussian blue staining of the kidney.

We further performed Prussian blue staining to evaluate the iron uptake in the liver, spleen, and kidney. The results suggested that iron uptake was strong in the spleen. A low level of iron uptake was seen in the liver. In contrast, no iron uptake was observed in the kidney (Figure 6).

### 2.6. Discussion

PSMA is an attractive target for PCa imaging due to the overexpression on almost all PCa cell surfaces [7]. In recent years, the development of PSMA-targeting image probes has been a major area of research. Most of previous studies employed anti-PSMA antibody for conjugating to imaging probes due to its well-established specificity [14,15,17]. However, the intact antibody allows the binding to the FcR on many circulating cells, endothelial cells, and liver cells and in turn might result in a low contrast ratio [18]. Therefore, small molecules markers, including antibody fragments, polypeptides, and aptamers, might be another option for the development of image probes.

Polypeptide is now becoming the research focus in PCa imaging probes development due to its high affinity to biomarkers and high penetration into tumor tissues. Wu* et al.* reported that CREKA peptide loaded contrast agent CREKA-dl-(DOTA-Gd)_4_ provided MRI contrast enhancement of PCa xenografts in mice by targeting fibronectin [19]. In another study, Tan *et al.* synthesized CLT1 peptide loaded MRI contrast agents for targeting fibrin-fibronectin complexes, the following* in vivo* studies demonstrated CLT1 loaded contrast agents provided excellent MRI contrast enhancement in PCa tumor-bearing mice [20]. However, these two peptides allowed for stroma targeting, not for PCa cell targeting. In our study, CQKHHNYLC, a low molecular weight polypeptide that specifically binds to PSMA [21], is used for the synthesis of molecular MRI imaging probe. The polypeptide-SPIONs exhibited a smooth and uniform spherical morphology with a diameter of 600–800 nm. The size of SPIONs was larger than in previous studies, because we planned for the synthesis of dual modality contrast agent for MRI/ultrasound in the next stage. The large size of SPIONs might generate micron-sized bubble after filling with perfluorocarbon gas, resulting in resonant peaks within clinical diagnostic ultrasound frequency range.

*In vitro* binding studies demonstrated greater polypeptide-SPIONs uptake by LNCaP cells compared with the control groups. In* in vivo* MRI studies, LNCaP tumor-bearing mice injected with polypeptide-SPIONs showed significant decreases in T2 tumor signal intensity post-injection. Further quantitative analyses revealed that there was significant increase of RSE 6–12 h post-injection. None of the tumors were hemorrhagic in histological analyses, suggesting the signal reduction in tumors was caused by SPIONs uptake. Prussian blue staining demonstrated significant polypeptide-SPIONs uptake within the tumor, which was also correlated with RSE measurement. By contrast, we found neither significant T2 signal changes nor SPIONs deposits in the control groups. These results suggested that polypeptide-SPIONs might offer specific enhancement of PCa tumors *in vivo* by targeting PSMA.

Several other groups of researchers also focused on developing PSMA-targeting MRI contrast agents. Most of these studies employed J591 antibody to conjugate with SPIONs for *in vitro* studies. Bates *et al.* reported that J591-SPIONs could enhance T2 signal of LNCaP cells* in vitro*. In comparison, no significant enhancement was observed in DU145 (PSMA negative) cells and LNCaP cells with non-targeted SPIONs [14]. In a recent research, Tse *et al.* demonstrated the J591-SPIONs led to increased LNCaP cell uptake of iron in *in vitro* studies. The researchers further injected J591-SPIONs into three LNCaP tumor-bearing mice; they found that there was enhancement of LNCaP tumor 2 and 24 h post-injection of J591-SPIONs [22]. In this work, polypeptide was employed to conjugate with SPIONs and the correlation between MRI enhancement, SPIONs concentration and MRI scan interval was studied systematically. Our results showed MRI enhancement increased with polypeptide-SPIONs concentration. The highest RSE was observed 6 h post-injection with an Fe_3_O_4_ concentration of 0.240 mg/mL. These results might be helpful for the further synthesis and evaluation of dual modality PSMA targeting contrast agent for MRI/ultrasound.

There are also limitations of our study. Firstly, the SPIONs with larger sizes are more likely to be broken down by macrophages compared with small SPIONs, and, thus, be removed from the blood stream so that they might have a comparably short blood half-life [23]. The results of our study also demonstrated the nonspecific SPIONs uptake in reticuloendothelial system. Further studies are needed to investigate the* in vivo* stability and bio-distribution of the SPIONs. In addition, subcutaneous tumor xenograft was employed for* in vivo* MRI studies. Therefore, these tumors might not reflect what occurs within the prostate gland. An orthotopic transplantation model should be established in further studies for better bridging of the gap between subcutaneous xenograft model and human studies.

## 3. Materials and Methods

### 3.1. Materials

Poly (vinyl alcohol) (PVA, 99% *M*w = 30,000–70,000) and PLGA [poly(lactic-co-glycolic acid) lactide:glycolide = 75:25, *M*w = 4000–15,000] were purchased from Sigma–Aldrich Company (Shanghai, China). *N*-Hydroxysuccinimide (NHS) and *N*-(3-dimethylaminopropyl)-*N*'-ethylcarbodiimide hydrochloride (EDC) were purchased from Acros Organics Company (Geel, Belgium). Carboxyl-functionalized Fe_3_O_4_ nanoparticles with a mean diameter of 30 nm were purchased from Huier Nano Technology Co., Ltd. (Henan, China). PSMA-targeting polypeptide (CQKHHNYLC, C_1_–C_9_ disulfide) was obtained from Beijing SciLight Biotechnology Co., Ltd. (Beijing, China).

### 3.2. Preparation of Polypeptide-PLGA

EDC (5 mg) and NHS (5 mg) were added to a PLGA (100 mg) solution in 10 mL of Dimethyl sulfoxide. The solution was stirred at room-temperature for 20 min, followed by the adding 2 mg of polypeptide to the solution. The mixture was stirred for 4 h at room temperature. Then, the polypeptide-PLGA was dialyzed against deionized water with a dialysis bag (Molecular Weight Cut Off = 3500 Da) for 1 day to remove EDC, NHS, and any residual, non-cross-linked polypeptide. Water in the sample was then removed by freeze-drying and kept at −4 °C for further use.

### 3.3. Preparation of Polypeptide-SPIONs

The preparation of the polypeptide-SPIONs was performed using a double emulsion method. Briefly, 0.6 mL of Fe_3_O_4_ nanoparticles (10 mg/mL) and 0.2 mL of 4% NH_4_HCO_3_ were added in polypeptide-PLGA polymer organic solution (50 mg polypeptide-PLGA in 2 mL methylene chloride), and the mixture was emulsified by sonication for 60 s. Free Fe_3_O_4_ nanoparticles from the formulations were removed using the magnetic isolation method [24]. Subsequently, 5 mL of cold PVA solution (5%, *w*/*v*) was added to this initial emulsion, and the mixture was homogenized for 5 min. The resulting double emulsion was diluted in 10 mL aqueous dimethylcarbinol (2%, *w*/*v*) under mechanical stirring at room temperature overnight. After centrifugation, the supernatant was discarded and the precipitate was washed with deionized water. The process of centrifugation and washing was repeated three times. Finally, after washing, the polypeptide-SPIONs were freeze-dried for further use. Non-targeted SPION swere prepared similarly, without the addition of polypeptide.

### 3.4. Characterization of Polypeptide-SPIONs

The mean diameter and size distribution of polypeptide-SPIONs was analyzed using a Malvern Zetasizer Nano ZS ZSP (Malvern Instrument, Worcestershire, UK). An inverted microscope (Nikon, TS100, Tokyo, Japan) was used to study the morphologies of polypeptide-SPIONs. The magnetization property was evaluated using a vibrating sample magnetometer (VSM, Model 7410, Lake Shore Cryotronics, Inc., Westerville, OH, USA).

### 3.5. In Vitro MRI of Polypeptide-SPIONs

*In vitro* MRI was performed using a 0.5T Meso MRI (Niumag MesoMR-60, Shanghai, China). The polypeptide-SPIONs with different concentration (Fe_3_O_4_: 0.060, 0.030, 0.015, 0.007 and 0.0045 mg/mL) were scanned and deionized water was served as controls. T2 weighted images were acquired with the following parameters: repetition time/echo time = 2500/100 ms; field of view = 60 × 60 mm; slice thickness = 3 mm; matrix size = 259 × 192. For transverse relaxation rate measurement, Carr–Purcell–Meiboom–Gill pulse sequence (SF = 23.311 MHz, P90 = 19 µs, P180 = 34 µs, TD = 103,430, SW = 100 KHz, TR = 1000 ms, RG1 = 20, RG2 = 3, NS = 4, NECH = 1000, TE = 1.034 ms) was performed. Transverse relaxation rate (*R*_2_) was calculated using the formula *R*_2_ (s^−1^·mM^−1^) = (1/T2s − 1/T2c)/*C*, where T2s is the relaxation time with polypeptide-SPIONs and T2c is the relaxation time without polypeptide-SPIONs, *C* is the concentration of polypeptide-SPIONs.

### 3.6. Cell Lines

PSMA-positive LNCaP cells and PSMA-negative PC3 cells were procured from American Type Culture Collection (ATCC, Rockville, MD, USA). All cells were grown in a 37 °C, 5% CO_2_ incubator using F-12 media (Invitrogen Life Technologies, Gaithersburg, MD, USA) supplemented with 10% fetal bovine serum (Invitrogen Life Technologies).

### 3.7. In Vitro Binding Assay

Prussian blue staining was employed to determine whether there was specific uptake of SPIONs by PCa cells. The cells were seeded in 24-well plates and incubated with polypeptide-SPIONs (Fe_3_O_4_, 0.060 mg/mL) or non-targeted SPIONs (Fe_3_O_4_, 0.060 mg/mL) for 12 h. After incubation, cells were washed three times in phosphate-buffered saline (PBS) to remove any free SPIONs, fixed with 4% paraformaldehyde for 20 min, washed with distilled water two times, acted with Perls reaction liquid (4% potassium ferrocyanide/6% HCl, 50:50 (*v*/*v*)) for 30 min, then rinsed and counterstained. The intracytoplasmic blue granules, which suggested cellular uptake of SPIONs (Figure 3A), were observed under a light microscope (Olympus IX51; Olympus, Tokyo, Japan) at 200× magnification.

### 3.8. Animal Model

All animal experiments were approved by the Institutional Animal Experimental Committee of Xinhua Hospital Affiliated to Shanghai Jiaotong University School of Medicine issued on 03 March 2014 (identification number: XHEC-D-2014-004) and performed in accordance with the recommendations of the National Institutes of Health Guide for the Care and Use of Laboratory Animals (Bethesda, MD, USA). Nude mice (male Balb/c mice, weighing between 25 and 30 g) aged 4–6 weeks were obtained from Slac Laboratory Animal Co., Ltd. (Shanghai, China). For creation of PCa subcutaneous xenograft model, mice were inoculated with 5 × 10^6^ cells/100 µL F12 combined with 100 µL Matrigel (BD Biosciences, Bedford, MA, USA) in the left flank. After implantation, mice were observed three times per week. Tumor size was measured in three dimensions (length, width, and height) with a digital caliper, and tumor volume was calculated using the formula for ellipsoid,* i.e.*, 0.52 × height × length × width. The mice qualified for MRI studies when tumor volume reached 0.5 mL.

### 3.9. In Vivo MRI Study

The tumor-bearing mice were anesthetized with ketamine (80 mg/kg), xylazine (3 mg/kg) and acepromazine (2 mg/kg) via intraperitoneal injection. Five mice were injected with each dose of polypeptide-SPIONS or non-targeted SPIONs. The route of injection was intravenous via retro-orbital vein. For the experiment group, LNCaP tumor-bearing mice were injected with 50 µL polypeptide-SPIONs with different concentrations (Fe_3_O_4_, 0.240 mg/mL, *n* = 5; Fe_3_O_4_, 0.120 mg/mL, *n* = 5; Fe_3_O_4_, 0.060 mg/mL, *n* = 5; Fe_3_O_4_, 0.030 mg/mL, *n* = 5). For the control group, LNCaP tumor-bearing mice and PC3 tumor-bearing mice were injected with 50 µL non-targeted SPIONs (Fe_3_O_4_, 0.240 mg/mL, *n* = 5) and polypeptide-SPIONs (Fe_3_O_4_, 0.240 mg/mL, *n* = 5), respectively. All* in vivo* MRI studies were performed before and 2, 6, and 12 h post-injection using a clinical 3.0 T whole-body MRI system (Signa HDxt, GE Healthcare, Milwaukee, WI, USA) with an eight-channel quadrature coil for signal reception. For each mouse, following localization scout scans, T2-weighted images were acquired with the following parameters: repetition time/echo time = 3620/123 ms; slice thickness = 2 mm; field of view = 180 × 180 mm^2^; matrix size = 512 × 512. After MRI, mice were euthanized with CO_2_, tumor tissues and other organs were harvested for histological analyses.

### 3.10. In Vivo MRI Image Analysis

After MRI evaluations, all images were interpreted by one radiologist. In order to determine whether there was T2 signal reduction of tumor tissue and other organs after the injection of SPIONs, the post-contrast MRI images were reviewed with pre-contrast images displayed side-by-side for comparison. The quantification analyses of MRI images were performed using an efilm work station (Merge Healthcare, Chicago, IL, USA). Relative signal enhancement (RSE) was employed to study the T2 signal change of tumors following SPIONs injection. For RSE calculations, *S*_mean_ was measured in tumor lesion and back muscle adjacent to the tumor. RSE was calculated using signal intensity measurements before SPIONs injection (*S*_mean_ pre) and post-injection (*S*_mean_ post) according to the following formula [25]: RSE (%) = 100 × [1 − (*S*_mean_ post in tumor/*S*_mean_ post in muscle)/(*S*_mean_ pre in tumor/*S*_mean_ pre in muscle)].

### 3.11. Histological Analysis

The harvested tissues were fixed with 4% paraformaldehyde, embedded and sectioned at a 5 µm thickness. For immunohistochemical staining, the sections were incubated in 3% H_2_O_2_/MeOH for 30 min to block endogenous peroxidase activity and then reacted with primary antibody against PSMA (ab19071, Abcam, Cambridge, MA, USA) at 4 °C overnight. Subsequently, the sections were washed with PBS and incubated with secondary antibody (Abcam, ab150115). The enzymatic reaction was developed in a freshly prepared solution of diaminobenzidine (0.5 mg/mL; Sigma, Deisenhofen, Germany) and 0.01% hydrogen peroxide in water. For Prussian blue staining, the sections were rinsed in distilled water and then incubated in Perls reaction liquid for 30 min and then rinsed and counterstained. The microscopic images (×100) with highest deposition of blue-stained foci were processed and analyzed with ImageJ software (National Institutes of Health, Bethesda, MD, USA). The area of Prussian blue deposition was automatically outlined and then measured by Image J software (National Institutes of Health, Bethesda, MA, USA). The Prussian blue labeling density was calculated by (Prussian blue staining area in pixels/total area in pixels) × 100.

### 3.12. Statistical Analysis

Differences between several groups were analyzed using one-way ANOVA and LSD post comparison tests. Pearson correlation coefficient was calculated to assess whether there was a correlation between RSE and Prussian blue labeling density. *p* values less than 0.05 were considered significant, and all statistical analyses were performed using Statistical Package for the Social Sciences (SPSS, Chicago, IL, USA).

## 4. Conclusions

In conclusion, we have developed polypeptide-SPIONs, which can enhance MRI imaging* in vivo* by targeting PSMA-overexpressing cells. Our results suggested that the negative contrast of PCa was related to the concentration and the time point of MRI scan. In this study, the optimal MRI scan interval was 6 h post-injection with a Fe_3_O_4_ concentration of 0.240 mg/mL for PCa subcutaneous xenograft models.

## Supplementary Materials

Supplementary materials can be found at http://www.mdpi.com/1422-0067/16/05/9573/s1.

## Abbreviations

PSMA: prostate specific membrane antigen
PCa: prostate cancer
MRI: magnetic resonance imaging
SPIONs: superparamagnetic iron oxide nanoparticles
PVA: Poly (vinyl alcohol) was used as stabilizing agent
PLGA: poly(lactic-co-glycolic acid) was used for the preparation of SPIONs
NHS: *N*-Hydroxysuccinimide was used as condensation agent
EDC: *N*-Hydroxysuccinimide; *N*-(3-Dimethylaminopropyl)-*N*'-ethylcarbodiimide hydrochloride was used as condensation agent
VSM: vibrating sample magnetometer was used for the characterization of the magnetization property of SPIONs
PBS: phosphate-buffered saline
RSE: relative signal enhancement was used for quantitative evaluation of negative contrast of MRI images following SPIONs injection
