# Supplementary material for: In Vivo Molecular MRI Imaging of Prostate Cancer by Targeting PSMA with Polypeptide-Labeled Superparamagnetic Iron Oxide Nanoparticles
Source: Int J Mol Sci. 2015 Apr 28;16(5):9573–87. doi: 10.3390/ijms16059573 (PMC4463605; doi:10.3390/ijms16059573)
Supplement: Supplementary file 1 [file ijms-16-09573-s001.pdf]

Supplementary Information

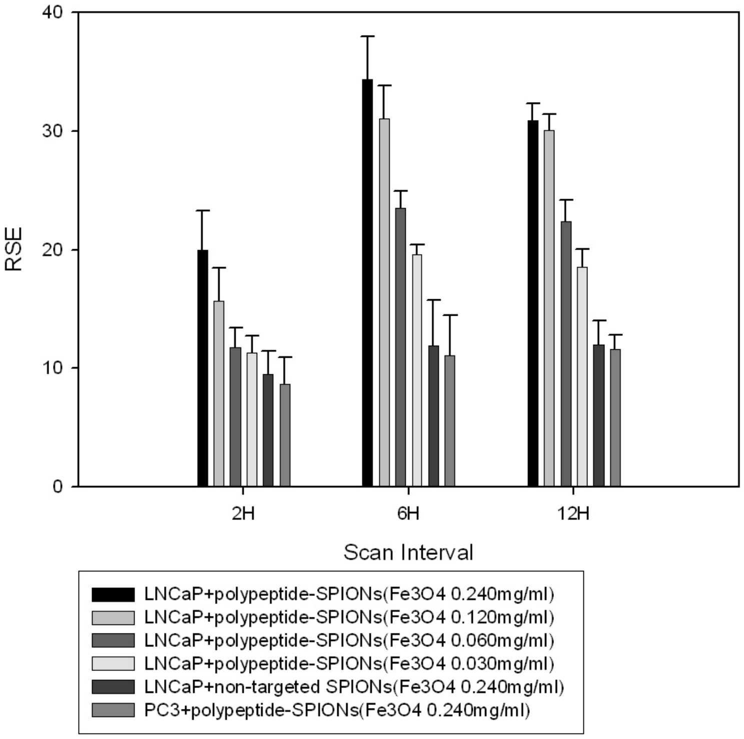

Figure S1. RSE measurements in LNCaP tumor-bearing mice injected with polypeptide-SPIONs, non-targeted SPIONs and PC3 tumor-bearing mice injected with polypeptide-SPIONs.

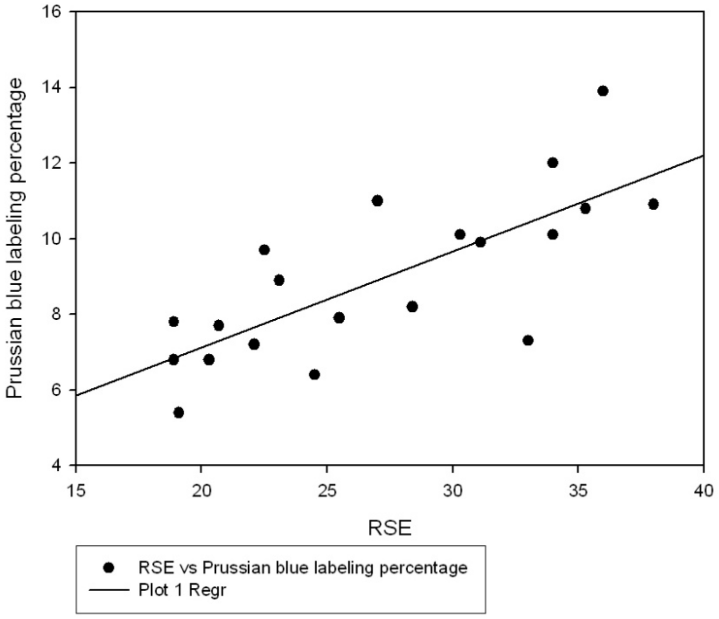

Figure S2. Correlation between RSE and Prussian blue labeling density in LNCaP tumor-bearing mice injected with polypeptide-SPIONs.
